# General Health (GHQ-28/CGHQ-28) and Psychosocial Risks (COPSOQ-ISTAS21) in Prehospital Emergency Professionals: A Pre-COVID-19 Cross-Sectional Study in Southern Spain

**DOI:** 10.3390/healthcare14010041

**Published:** 2025-12-23

**Authors:** José Antonio Morales-García, Francisco Manuel Ocaña-Peinado, Víctor Javier Costela-Ruiz, Elvira De Luna-Bertos, Javier Ramos-Torrecillas, Olga García-Martínez

**Affiliations:** 1 061 Health Emergency Center, Andalusian Health Service, Junta de Andalucía, 18016 Granada, Spain; joseantonio.morales.epes@juntadeandalucia.es; 2 Biomedical Group (BIO277), Department of Nursing, Faculty of Health Sciences, University of Granada, 18071 Granada, Spain; vircoss@ugr.es (V.J.C.-R.); elviradlb@ugr.es (E.D.L.-B.); ogm@ugr.es (O.G.-M.); 3 Department of Statistics and Operations Research, School of Pharmacy, University of Granada, 18011 Granada, Spain; fmocan@ugr.es

**Keywords:** psychosocial risks, occupational disease, working conditions, emergency medical services

## Abstract

**Highlights:**

**What are the main findings?**

- Cross-sectional, pre-COVID-19 (July–September 2019) study of prehospital emergency professionals (061 Health Emergency Center, Granada, Spain; n = 51) assessing general health and psychosocial risks.
- Uses GHQ-28/CGHQ-28 and COPSOQ-ISTAS21 to quantify self-perceived health, chronic strain, and exposure across key dimensions (psychological demands, double presence/work–family conflict, esteem, support/leadership).

**What are the implications of the main findings?**

- Despite good self-perceived health, chronic difficulties were common and exposure to adverse psychosocial dimensions was high—notably psychological demands, double presence, and esteem—across sex, shift type, and role.
- Actionable implications: Prioritize organizational measures to reduce demands and work–family conflict, strengthen leadership and peer support, and establish routine monitoring (GHQ-28 + COPSOQ-ISTAS21); the study provides a pre-COVID-19 baseline for future comparisons.

**Abstract:**

**Background:** Prehospital emergency professionals are exposed to high psychosocial demands that may impact their mental health, but pre-COVID-19 baseline data from Spanish services are scarce. This study aimed to assess the general health and psychosocial risk factors in a regional prehospital emergency service before the COVID-19 pandemic. **Methods:** We conducted a cross-sectional descriptive study (September–December 2019) including 51 physicians, nurses, and emergency medical technicians working at the 061 Health Emergency Center in Granada (Andalusia, Spain). General health and chronic problems were assessed with the Goldberg General Health Questionnaire (GHQ-28/CGHQ-28), and work-related psychosocial risks were evaluated using the COPSOQ-ISTAS21 questionnaire. Descriptive statistics, group comparisons, and exploratory Spearman correlations between health indicators and psychosocial dimensions were performed. **Results:** Most participants reported good self-perceived general health, but the chronic coding of the GHQ (CGHQ-28) indicated long-term difficulties mainly related to social dysfunction, somatic symptoms, and anxiety/insomnia. Exposure to unfavorable psychosocial risk was frequent, particularly in psychological demands, double presence (work–family conflict), and low esteem, with intermediate–unfavorable patterns in active job/development, insecurity, and social support/leadership. Exploratory correlations suggested that double presence was the psychosocial factor most consistently associated with chronic distress. **Conclusions:** In this pre-COVID-19 cohort of prehospital emergency professionals, good perceived general health coexisted with chronic psychological strain and high exposure to adverse psychosocial work factors. These findings support the need for organizational measures to reduce psychological demands and work–family conflict and to strengthen social support and leadership in prehospital emergency teams.

## 1. Introduction

The mission of prehospital health emergency services in Spain is to deliver urgent and emergency healthcare in a safe and effective manner, in addition to contributing to improving the health of the population. Teams must act rapidly in sometimes complex and high-pressure circumstances to stabilize clinically severe patients and enable their transfer to appropriate referral hospitals. Attended patients can suffer from acute myocardial infarction, cardiac arrest, severe traumatism, stroke, or severe respiratory failure, among other conditions, and their prognosis is strongly determined by the initial care delivered by the prehospital health emergency team [1].

Psychosocial risk factors refer to the conditions of employment, especially its organization, with a potentially negative effect on health via psychological and physiological mechanisms. In work settings, stress due to these factors can often produce exhaustion, with long-lasting or chronic effects that can impact on the performance of individuals and on their intention to quit, reducing job satisfaction and impairing quality of life [2]. Disorders related to occupational stress can predispose professionals to develop poor hygiene and health habits, affecting the safe and competent delivery of the care they provide and reducing its quality [3].

Beyond individual-level symptoms, occupational stress in healthcare and emergency services has been conceptualized within classic models such as Karasek’s Job Demand–Control (JDC) model, Siegrist’s Effort–Reward Imbalance (ERI) model, and the more recent Job Demands–Resources (JD-R) framework. These models posit that combinations of high psychological demands, low job control, low rewards (e.g., esteem, recognition, job security), and insufficient resources (e.g., social support, leadership quality, opportunities for development) increase the risk of mental health problems and chronic strain. The COPSOQ-ISTAS21 dimensions used in this study map onto these constructs, providing a structured assessment of demands, control, insecurity, esteem, and social support. Applying these frameworks to prehospital emergency work offers theoretical lenses through which patterns of psychosocial exposure and long-term health outcomes can be interpreted in an integrated manner [4,5,6].

Identifying this type of problem, which frequently goes unnoticed in primary care, may contribute to improving the diagnostic and therapeutic performance of healthcare professionals, increasing the quality and cost-effectiveness of the services that they deliver. Psychosocial hazards may silently erode decision-making, recovery between shifts, and team climate, ultimately impacting safety and care quality in time-critical settings [7,8]. Previous studies from other European emergency medical services have reported high psychological demands, work–family conflict, and stress-related outcomes among ambulance personnel and emergency nurses [9]. However, pre-COVID-19 empirical data on the general health and psychosocial risk profiles in Spanish prehospital emergency teams are scarce and fragmented, and most available studies come from hospital or primary care settings [10,11]. In particular, to our knowledge no previous work has simultaneously combined the GHQ-28 and its chronic coding (CGHQ-28) with the COPSOQ-ISTAS21 in this context, which limits our understanding of how specific psychosocial work factors translate into both acute and chronic psychological distress in prehospital emergency services.

By providing an integrated baseline assessment of general health (GHQ-28/CGHQ-28) and psychosocial exposures (COPSOQ-ISTAS21) in an emergency center shortly before the COVID-19 pandemic, our study contributes to this evidence base and facilitates future cross-country and pre–post-pandemic comparisons. The objective of this study was to assess general health (GHQ-28/CGHQ-28) and work-related psychosocial risks (COPSOQ-ISTAS21) among professionals at the 061 Health Emergency Center (061HEC) in Granada, Spain (pre-COVID-19, September–December 2019). In addition to describing the distribution of these variables, the study establishes a pre-pandemic baseline for a Southern European prehospital emergency service and jointly applies both GHQ-28/CGHQ-28 and COPSOQ-ISTAS21 within the same cohort. This combined approach allows us to explore the apparent paradox between good self-perceived health and high levels of chronic strain, a phenomenon that has been increasingly recognized in high-risk professions.

## 2. Materials and Methods

### 2.1. Design, Sample, and Setting

This cross-sectional descriptive study used questionnaires to gather information from 54 professionals in the 061HEC between September and December 2019. The inclusion criterion was to be in active employment as a physician, nurse, or emergency medical technician (EMT; corresponding to Spanish “técnicos en emergencias sanitarias”, who typically serve as ambulance technicians/drivers) with at least two years’ work experience in the same position, whether contracted or on the permanent staff. Exclusion criteria were the diagnosis of an anxiety-related mental disorder or the personal experience of a traumatic event unrelated to work (e.g., divorce, loss of a close relative, etc.) in the previous six months. The accessible population comprised n = 54. All were informed about the study during staff meetings and via internal communication channels and were invited to participate. Participation was strictly voluntary, unrelated to performance evaluation, and with no consequences for declining or withdrawing. Of these, 51 professionals met the eligibility criteria and completed all instruments (response rate 51/54 = 94.4%; 95% CI 84.9–98.1).

Given the exploratory, single-center design, no a priori sample size calculation was performed. Instead, the target population was defined as the full workforce of the 061HEC, and the final sample represents almost the entire census of this center (51/54 professionals). This sample size allows us to obtain reasonably precise prevalence estimates for GHQ-28/CGHQ-28 and COPSOQ-ISTAS21 scores but limits the statistical power to detect small between-group differences, which are therefore interpreted with caution.

### 2.2. Instruments

Questionnaires were self-administered and completed during rest periods with the presence of the principal researcher to resolve any doubts. An ad hoc questionnaire was used to gather data on age, sex, marital status, type of work shift, and profession.

The Goldberg General Health Questionnaire (GHQ-28), validated for Spanish populations, was used to assess self-perceived general health status. It comprises 28 items divided into four subscales (Somatic Symptoms, Anxiety and Insomnia, Social Dysfunction, and Depression), rated on a four-point Likert scale [12]. For the GHQ-28, we used the standard binary scoring (0–0-1-1) and a 5/6 cut-off to identify probable cases, as recommended in Spanish validation studies, which have shown good sensitivity, specificity, and internal consistency [12,13,14,15]. The chronic coding (CGHQ-28; 0-1-1-1) and a 12/13 cut-off were used to identify long-term or chronic problems, following previous work [15].

Psychosocial work factors were assessed with the Spanish COPSOQ-ISTAS21 questionnaire, which measures six dimensions: Psychological Demands, Active Job/Development, Insecurity, Social Support/Leadership Quality, Double Presence, and Esteem. The Spanish version has demonstrated good reliability and validity in occupational settings [16,17,18].

Raw scores were calculated for each dimension following the Spanish ISTAS21 manual. In risk dimensions (e.g., Psychological Demands, Insecurity, Double Presence), higher scores reflect greater exposure, whereas in resource dimensions (Active Job/Development, Social Support/Leadership, Esteem) higher scores reflect more favorable job resources. For interpretation, raw scores were classified into three exposure categories favorable (psychosocial exposure more favorable for health), intermediate (psychosocial events generating moderate emotional disorders), and unfavorable (psychosocial exposure requiring immediate intervention), according to the reference cut-offs provided in the Spanish ISTAS21 manual.

### 2.3. Data Gathering and Ethical Considerations

Participation was voluntary and required the signing of informed consent. Information was gathered in full compliance with data protection legislation, and the study was approved by the Regional Research Ethics Committee (code: 1602-N-17).

### 2.4. Data Analysis

SPSS version 27.0 (IBM SPSS, Armonk, NY, USA) was used for statistical analyses. Quantitative variables were expressed as means with standard deviations and qualitative variables as percentages. The normal distribution of variables was verified with the Shapiro–Wilk test. The association among qualitative variables was analyzed using the chi-square test (with Fisher’s correction in the case of 2 × 2 tables), and each category of quantitative variables was analyzed by means of Student’s *t*-test or Mann–Whitney U test (cases with two samples), or an ANOVA or the Kruskal–Wallis test (cases with multiple samples); *p* ≤ 0.05 was considered significant in all tests.

Between-group comparisons according to sociodemographic (sex, marital status) and professional variables (role, shift type) were planned a priori as exploratory analyses to identify potentially vulnerable subgroups within a shared organizational shift system; therefore, they were interpreted descriptively and with caution given the small single-center sample. Because of the limited sample size and number of outcome events, we did not fit multivariable models to adjust for potential confounders such as age or job tenure; instead, these variables were summarized descriptively and considered when interpreting GHQ-28/CGHQ-28 and COPSOQ-ISTAS21 patterns. Spearman’s rank correlation coefficients (ρ) between GHQ-28/CGHQ-28 scores and COPSOQ-ISTAS21 dimensions were computed as exploratory measures of association and interpreted qualitatively as effect sizes. No additional multivariable modeling or formal effect size indices (e.g., Cohen’s d) were calculated to avoid overinterpreting underpowered contrasts.

## 3. Results

Out of the 54 professionals volunteering for the study, 51 (58.8% male) met the eligibility criteria (23 physicians, 16 nurses, and 12 EMTs), with a mean age of 49.72 ± 4.9 years; 62.7% of participants were married, 15.7% were separated, 11.8% had common-law partners, and 7.8% were single; they had a mean of 1.66 ± 0.95 children.

Almost all participants (98%) held a permanent position, with a mean of 19.74 ± 4.2 years in the 061HEC. All participants had rotating work shifts (morning–afternoon–night). More than half, n = 27 (52.9%), performed management tasks within the emergency team, n = 2 (3.9%) worked in coordination, and n = 22 (43.2%) in both (Table 1).

The global GHQ score was a mean of 3.43 ± 5.14 (cut-off point of 5/6, no case/case). Subscale scores were 1.41 ± 2.16 for Somatic Symptoms, 1.16 ± 1.79 for Anxiety and Insomnia, 0.71 ± 1.60 for Social Dysfunction, and 0.16 ± 0.64 for Depression. No significant differences were found in global or subscale scores as a function of sex, marital status, type of work shifts, or profession, as shown in Table 2. The score was 0 (better or the same as normal) for the Somatic Symptoms subscale in 56.9% of participants, for Anxiety and Insomnia in 60.8%, for Social Dysfunction in 74.5%, and for Depression in 92.2% (Table 3). The global score was 0 in 47.1% of participants.

The global CGHQ score was a mean of 15.14 ± 5.36 (cut-off point of 12/13, no case/case). The highest mean subscale score was for Social Dysfunction (6.25 ± 1.64), followed by Somatic Symptoms (4.16 ± 2.18) and Anxiety and Insomnia (3.84 ± 2.25), and the lowest score was for Depression (0.88 ± 1.58). No statistically significant differences were observed as a function of any study variable (Table 4). Considering 12/13 as the cut-offf point, 76.5% of participants were cases, i.e., with the presence of chronic health problems, and the highest subscale scores were for Social Dysfunction, which was 7 points in 72.5% of participants, followed by Somatic Symptoms, ≥4 points in 62.7%, and Anxiety and Insomnia, ≥4 points in 56.8%. In contrast, a score of 0 was obtained for the Depression subscale in 64.7% of participants (Table 3).

**Figure 1:**
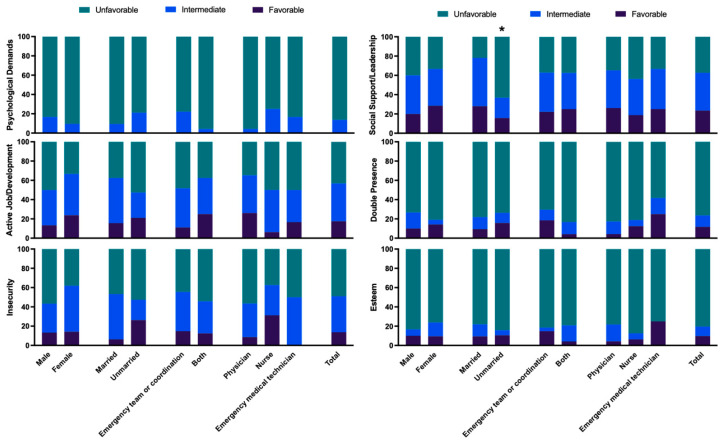
Percentage of workers in favorable, intermediate, and unfavorable exposure categories for each COPSOQ-ISTAS21 dimension. Bars represent the percentage of participants whose raw scores fall into the favorable, intermediate, or unfavorable bands defined by the Spanish ISTAS21 reference cut-offs for each dimension. * *p* ≤ 0.05.

Analysis of psychosocial risk factors revealed an unfavorable situation for >76.5% of participants in relation to three of the six dimensions, i.e., Psychological Demands, Double Presence, and Esteem (Figure 1); and an intermediate–unfavorable situation for >37.3% in Active Employment/Possibilities of Development, Insecurity, and Social Support/Leadership Quality. In relation to Psychological Demands, an unfavorable situation was reported by 86.3% of the professionals. No statistically significant differences were observed as a function of the sex, marital status, work shift type, or profession of the participants (Figure 1).

Results for the Active Employment/Possibilities of Development dimension were unfavorable in 43.1% of participants and intermediate in 39.2%. Results for Insecurity were unfavorable in 49.0% and intermediate in 37.3% No statistically significant differences were observed as a function of the sex, marital status, work shift type, or profession (Figure 1). Results for Social Support/Leadership Quality were unfavorable in 37.3% of participants and intermediate in 39.2%, observing statistically significant difference only between unmarried (21.63 ± 7.7) and married (26.25 ± 4.64) participants (Table 5), with the unmarried describing a less favorable situation (Figure 1). Results were unfavorable for Double Presence in 76.5% of participants and for Esteem in 80.4% (Figure 1), finding no significant differences by sex, marital status, shift type, or profession.

Spearman correlation analyses are presented in Supplementary Tables S1–S3. First, convergent validity between GHQ-28 and CGHQ-28 was supported by the pattern of correlations between equivalent subscales and total scores. Three of the four corresponding subscales showed moderate and statistically significant correlations (ρ = 0.491–0.568), whereas the Social Dysfunction subscales were more weakly related (ρ = 0.099; *p* = 0.487). The correlation between GHQ-28 and CGHQ-28 total scores was moderate-to-high (ρ = 0.552; *p* < 0.001). Internal correlations within each questionnaire were high (ρ = 0.392–0.886 for GHQ-28 and ρ = 0.291–0.871 for CGHQ-28), consistent with good internal coherence. Second, correlations between GHQ-28 subscales and COPSOQ-ISTAS21 dimensions were generally low and non-significant (ρ = −0.175 to 0.249; *p* > 0.05), indicating no strong association between general psychological distress and the psychosocial work factors assessed in this sample (Supplementary Table S2). Third, a similar pattern was observed for CGHQ-28, although small but statistically significant positive correlations emerged between Double Presence and the CGHQ-28 Anxiety–Insomnia subscale (ρ = 0.336; *p* = 0.016) and CGHQ-28 total scores (ρ = 0.312; *p* = 0.026), with a borderline association for Somatic Symptoms (ρ = 0.273; *p* = 0.053). Within COPSOQ-ISTAS21, theoretically expected relationships were confirmed, including an inverse correlation between Psychological Demands and Active Job Development and strong positive correlations between Active Job Development and Social Support/Leadership and between Social Support/Leadership and Esteem. Overall, these exploratory analyses highlight Double Presence (work–family conflict) as the psychosocial factor most clearly associated with chronic psychological distress in this cohort (Supplementary Tables S1–S3).

## 4. Discussion

In this pre-COVID-19 cohort of prehospital emergency professionals, we observed an apparent paradox: most participants reported good self-perceived general health according to the GHQ-28, while the chronic coding (CGHQ-28) revealed a high proportion of long-term problems, mainly in social dysfunction, somatic symptoms, and anxiety/insomnia. In parallel, COPSOQ-ISTAS21 scores showed a predominance of intermediate to unfavorable exposure in several psychosocial dimensions, particularly psychological demands, double presence, and low esteem. Together, these findings are consistent with the notion that chronic strain and adverse psychosocial conditions may have been common in this service before the COVID-19 pandemic.

The data for this study were collected before the COVID-19 pandemic, whose negative impact on the mental health of healthcare professionals has been well documented [19]. However, limited information is available on the general health status and psychosocial risks of Spanish healthcare professionals before the pandemic. Our results are broadly consistent with previous studies in emergency medical services and hospital settings, which have also reported high psychosocial demands, work–family conflict, and stress-related outcomes among physicians, nurses, and EMTs [10]. In Spanish nursing populations, similar patterns of chronic strain and sleep problems have been described, although most of this evidence comes from hospital and primary care rather than prehospital teams [20,21,22,23,24]. In 2021, the Andalusian regional government launched a humanization strategy that included improvement of the working conditions of healthcare professionals [25].

The GHQ results showed a good self-perceived general health status, especially in relation to Somatic Symptoms (1.41 ± 2.16) and Anxiety and Insomnia (1.16 ± 1.79). In the same line, studies of hospital nurses demonstrated acceptable levels of mental health [21,26]. A worse GHQ-28 score for general health perception was reported in a study of prehospital emergency physicians from Iran, which may be influenced by their younger mean age (31.91 ± 6.9 vs. 49.7 ± 4.8 yrs) and shorter history of experience/training (<10 yrs vs. ≈20 yrs) in comparison to the present participants [27]. Younger age and lesser experience have been associated with work-related stress and anxiety among healthcare professionals, whose psychological stress was found to be higher during their first two years in employment; it is possible that more experienced professionals may adapt better to stress at work, which may explain the general health perception of our study population [28,29]. Importantly, the study excluded professionals with a personal experience of a traumatic event unrelated to work during the previous six months, which can produce a significant increase in the GHQ score [30]. CGHQ-28 results showed that 76.5% of participants met the case threshold, indicating chronic health problems. One possible explanation, though untested, for the favorable self-perception of health in the 061 HEC professionals may relate to the progressive habituation to strain and the tendency to respond with “the same as normal” (0 points) in circumstances that, over time, have been integrated into everyday life despite being unpleasant or disturbing. This pattern may be tentatively interpreted as a form of “normalization of discomfort” in a high-risk profession. Over time, somatic complaints, sleep problems, and role overload could become part of everyday expectations and therefore be underreported in global self-perceived health [14,31]. However, psychological mechanisms such as habituation, coping, or resilience were not directly measured in this study and remain speculative hypotheses that should be examined in future research rather than taken as demonstrated processes.

At the same time, the relatively high mean age and long job tenure of this cohort, together with the exclusion of professionals with a recent non-work-related traumatic event, may have attenuated the levels of distress observed and partly contributed to the lack of marked differences between subgroups. Because of the limited sample size, we could not statistically control for age, tenure, or other potential confounders, so our between-group comparisons should be interpreted as exploratory and hypothesis-generating rather than confirmatory.

Among CGHQ subscales, the highest score was for Social Dysfunction, followed by Somatic Symptoms and Anxiety and Insomnia, while the lowest score was for Depression. These findings may reflect chronic difficulties in maintaining daily functioning, potentially associated with professional strain or burnout risk [32]. Anxiety in health professionals, with somatic manifestations related to sleeping problems, has been associated with the high emotional burden associated with a heavy workload, exposure to existing and emerging diseases, dealing with the suffering of people and their families, and shiftwork [33,34].

These results therefore reflect a paradox that has been repeatedly described among healthcare professionals: although they report a good perception of overall health, their long-term assessments reveal chronic problems related to social dysfunction, somatic symptoms, anxiety, and insomnia. Over time, somatic complaints, sleep problems, and role overload may be integrated into baseline expectations and therefore underreported in global self-perceived health (GHQ-28), while being captured by the chronic coding (CGHQ-28). In our cohort, this mechanism could explain why the mean GHQ-28 score remains low whereas 76.5% of participants meet the CGHQ-28 case threshold, suggesting that chronic strain has been internalized without necessarily being labeled as poor health [35].

No significant differences were found in any subscale in GHQ or CGHQ with respect to sex, marital status, shift type, or profession, which may be because all of these professionals work under the same conditions and therefore share the same effects, regardless of these characteristics [36].

Results obtained with the COPSOQ-ISTAS21 questionnaire revealed a predominance of scores indicating intermediate/unfavorable risk in all dimensions (Psychological Demands, Active Employment, Insecurity, Social Support and Quality in Leadership, Double Presence, and Esteem). This finding agrees with the assessment of chronic health problems obtained in the CGHQ, indicating that the work conditions and organization of prehospital emergency health professionals generate exposure over time to psychosocial risks that can undermine their health.

Scores indicating unfavorable risk were obtained for the dimensions Psychological Demands, Double Presence, and Esteem in more than half of participants, regardless of their profession (physician, nurse, or EMT), and the sole statistically significant difference was observed between married and unmarried participants in Esteem, although this assessment remained unfavorable in both groups. Similar results were published by Fernández-Prada et al. [37], who observed a high risk in these categories in 78% of their sample of emergency physicians-in-training. A high risk assessment in all dimensions was also reported among nurses employed in hospital emergency care [10].

Scores indicating intermediate risk were obtained for the dimensions Active Employment/Possibilities of Development, Insecurity, and Social Support/Leadership Quality; the only statistically significant difference among participants was in Social Support/Leadership Quality with an unfavorable risk assessment in unmarried professionals versus an intermediate risk assessment in those who were married. This might be related to known stress generators such as limited autonomy at work, low remuneration, high care load in a very complex setting, and a lack of social support [10]. Living accompanied might therefore be a possible protective factor, given previous findings of the increased anxiety and work stress in health professionals living alone in comparison to those living with others [38].

All the data indicate that although healthcare professionals perceive themselves as having good overall health, the presence of somatic symptoms, social dysfunction, anxiety, and insomnia reveals a sustained impact of occupational stress on their long-term well-being. This duality may reflect an adaptive pattern sometimes interpreted as the resilience capacity of this professional group, who tend to maintain a positive perception of their health despite the accumulation of physical and emotional strain associated with work conditions.

Beyond descriptive comparisons, the exploratory correlation analyses provided additional insight into the relationships between general health, chronic problems, and psychosocial work factors (Supplementary Tables S1–S3). First, moderate-to-high correlations between parallel GHQ-28 and CGHQ-28 subscales and total scores supported the convergent validity and internal coherence of the chronic coding in this occupational context. This suggests that the CGHQ-28 does not introduce a different construct but rather captures the chronic counterpart of the same dimensions of somatic symptoms, anxiety–insomnia, social dysfunction, and depression. Second, correlations between global distress indices and COPSOQ-ISTAS21 dimensions were generally small, with only the “Double Presence” factor showing consistent positive associations with the chronic anxiety/insomnia subscale and the overall CGHQ-28 score, and a borderline association with somatic symptoms. In line with our descriptive profile and previous findings in emergency and hospital settings [10,37], this pattern indicates that the combined load of paid work and unpaid caregiving may represent a particularly salient pathway linking psychosocial strain to chronic health problems in prehospital emergency services, whereas other job demands and resources may exert more diffuse or indirect effects that our sample was underpowered to detect.

The elevated psychosocial risks identified (particularly in terms of psychological demands, double presence, and low esteem) underscore the decisive role of organizational determinants in healthcare workers’ mental health. These results suggest priorities for mitigating psychological strain and promoting supportive leadership dynamics. The observed pattern of high demands and constrained resources is consistent with the profiles postulated by the JDC, ERI, and JD-R theoretical frameworks [4,5,6]. Within these models, such imbalances are expected to generate chronic strain, somatic symptoms, and sleep problems, which align with the CGHQ-28 pattern of social dysfunction, somatic complaints, and anxiety/insomnia in our participants. Interpreting our findings through these models underscores that the key levers for prevention lie in work organization, shift design, recognition practices, and supportive leadership, rather than in individual resilience alone.

Policy alignment and implementation pathways

Our 2019 snapshot provides a pre-COVID-19 baseline against which post-pandemic trajectories in prehospital emergency teams can be interpreted. Current international frameworks on psychosocial risk management at work (such as ISO 45003:2021, the WHO/ILO Guidelines on Mental Health at Work, and the EU Strategic Framework on OSH 2021–2027) [39,40,41] place the emphasis on the primary prevention, organizational risk assessment, and routine monitoring of psychosocial hazards. The high levels of psychological demands, double presence, and low esteem observed in our cohort indicate that these domains are particularly relevant targets when implementing such frameworks in emergency medical services. In parallel, the recognition of burnout as an occupational phenomenon in ICD-11 and the recent syntheses on moral distress and moral injury in healthcare workers [42,43] underline the need to complement individual-level interventions with organizational measures that improve workload, leadership, and ethical support.

Strengths and limitations

This study has several strengths. We used psychometrically validated instruments (GHQ-28, CGHQ-28, and COPSOQ-ISTAS21) to assess both general health and a broad range of psychosocial risk factors. The survey covered the entire staff of a regional prehospital emergency service, minimizing selection related to voluntary participation within the service. In addition, data were collected shortly before the COVID-19 pandemic, providing a unique pre-pandemic baseline for subsequent comparisons of emergency medical services.

However, it is important to consider certain limitations. First, the cross-sectional design precludes causal inference regarding the relationships between psychosocial exposures and health outcomes; therefore, our findings should be interpreted as associative and exploratory. Second, the sample was restricted to a single public emergency medical service in Andalusia, with a relatively small number of professionals, which limits the generalizability of the results to other regions, countries, and service models. Third, all variables were self-reported and thus subject to reporting and common-method bias; for instance, social desirability or professional identity may have led to underreporting of distress in global health items while over- or underestimating specific symptoms. Fourth, we excluded professionals who had experienced a recent non-work-related traumatic life event to avoid acute confounding, which may have led to a slight underestimation of distress levels and reduces comparability with studies that did not apply similar criteria. Fifth, we did not collect detailed contextual organizational indicators, such as objective workload intensity, number and type of emergency calls, staffing levels, or individual shift patterns, nor did we statistically adjust the analyses for potential confounders (e.g., age, tenure, contract type, comorbidities); consequently, residual confounding cannot be ruled out. Sixth, apart from the confidence interval reported for the participation rate, we did not systematically report confidence intervals or formal standardized effect size indices (e.g., Cohen’s d) for prevalence estimates and between-group comparisons, focusing instead on descriptive estimates and correlation coefficients as qualitative indicators of association. This further reinforces the exploratory, hypothesis-generating nature of our analyses.

Taken together, these limitations indicate that the results should be regarded as a descriptive snapshot of a single regional service and as exploratory evidence that requires replication and extension in larger, multi-center longitudinal cohorts, incorporating organizational metrics and more robust adjustment for potential confounders.

Strategic Lines for Human Resource Management and Future Work

Based on this pre-COVID-19 baseline and in line with the above policy frameworks, several strategic lines for human resource management and future research can be outlined:

- Primary prevention bundles mapped to ISO 45003 domains (workload/tempo, role clarity, job control, recognition/esteem, social support/leadership) and to relevant COPSOQ-ISTAS21 dimensions; co-design with frontline staff and evaluate with repeated GHQ-28/CGHQ-28 + COPSOQ panels.
- Shift design and fatigue risk management (limits to consecutive nights, protected recovery windows, predictable rosters) to reduce psychological demands and double presence; track sleep/insomnia, sickness absence, and safety incidents as leading indicators.
- Leadership and peer support: Manager training in supportive supervision; routine post-incident debriefing; confidential help-seeking pathways; monitor uptake and perceived leadership support (COPSOQ).
- Work–family conflict (double presence): Flexible scheduling and access to caregiving support; monitor equity by sex/caregiving status in line with EU OSH priorities.
- Ethical climate and moral injury: Include validated measures of moral distress/injury in longitudinal monitoring; test team-level ethics/reflection rounds for effects on anxiety/depression and CGHQ cases [43].
- Implementation science: Use RE-AIM/CFIR (Reach, Effectiveness/Efficacy, Adoption, Implementation, Maintenance/Consolidated Framework for Implementation Research) logic to report reach, adoption, fidelity, and maintenance alongside effectiveness, feasibility, and costs to support scalable roll-out across emergency medical services bases.

## 5. Conclusions

In this pre-COVID-19 cohort of prehospital emergency professionals, most participants reported good self-perceived general health, while the chronic coding of the GHQ (CGHQ-28) revealed a high proportion of long-term problems, mainly related to social dysfunction, somatic symptoms, and anxiety/insomnia. At the same time, exposure to psychosocial risks was frequent, particularly regarding psychological demands, double presence (work–family conflict), and low esteem.

Taken together, these findings suggest that chronic strain and unfavorable psychosocial conditions were already present and may have been partly normalized within this prehospital emergency service before the COVID-19 pandemic. Given the cross-sectional, single-center, and exploratory nature of the study, the results should be interpreted with caution. Future multi-center and longitudinal research, integrating organizational indicators and more robust adjustment for confounders, is needed to confirm these patterns and to better inform the design of interventions aimed at protecting the mental health of prehospital emergency professionals.

## Figures and Tables

**Table 1:** Description of the sociodemographic variables of professionals of the 061 Health Emergency Center.

	n	%
**Marital status**		
Single	4	7.8
Married	32	62.7
Separated	8	15.7
Widower	1	2
Common-law partners	6	11.8
*Total*	51	100
**Sex**		
Male	30	58.8
Female	21	41.2
*Total*	51	100
**Profession**		
Physician	23	45.1
Nurse	16	31.4
EMT ^a^	12	23.5
*Total*	51	100
**Employment status**		
Permanent	50	98
Substitute	1	2
*Total*	51	100
**Type of work shift**		
Morning/evening/night	51	100
**Professional role**		
Emergency team	27	52.9
Coordination	2	3.9
Both	22	43.2
*Total*	51	100
	**Media**	**SD**
**Age**	49.72	4.9
**Children**	1.66	0.95
**Length of service**	19.74	4.2

^a^ EMT: emergency medical technician.

**Table 2:** Scores of the perceived general health status (GHQ-28) of subscales, and comparison among variables.

			Sex		Marital Status		Professional Role		Profession		
	Total		Male	Female	Married	Unmarried	Emergency Team or Coordination	Both	Physician	Nurse	EMT ^a^
	n	51	30	21	32	19	27	24	23	16	12
***Somatic*** ***Symptoms***	**Mean (SD)**	1.41 (2.16)	0.97 (1.85)	2.05 (2.44)	1.22 (2.04)	1.74 (2.35)	1.48 (2.23)	1.33 (2.12)	1.13 (1.91)	2.44 (2.76)	0.58 (1)
	***p***	-	-	0.107	-	0.650	-	0.802	-	0.174	0.514
***Anxiety and*** ***Insomnia***	**Mean (SD)**	1.16 (1.79)	0.93 (1.46)	1.48 (2.18)	0.97 (1.62)	1.47 (2.06)	1.22 (1.89)	1.08 (1.72)	0.91 (1.53)	1.69 (2.24)	0.92 (1.56)
	***p***	-	-	0.663	-	0.506	-	0.932	-	0.465	1
***Social*** ***Dysfunction***	**Mean (SD)**	0.71 (1.60)	0.6 (1.43)	0.86 (1.85)	0.66 (1.43)	0.79 (1.90)	0.67 (1.64)	0.75 (1.59)	0.57 (1.34)	1 (2.07)	0.58 (1.44)
	***p***	-	-	0.608	-	0.929	-	0.990	-	1	1
***Depression***	**Mean (SD)**	0.16 (0.64)	0.03 (0.18)	0.33 (0.97)	0.06 (0.25)	0.32 (1)	0.04 (0.19)	0.29 (0.91)	0.13 (0.46)	0.31 (1.01)	0(0)
	***p***	-	-	0.145	-	0.531	-	0.232	-	0.782	0.350
***Global Score***	**Mean (SD)**	3.43 (5.14)									

^a^ EMT: emergency medical technician.

**Table 3:** Distribution (%) of subscale scores (0–7) for the GHQ-28 and its chronic form (CGHQ-28) in prehospital emergency professionals (n = 51).

	Somatic Symptoms		Anxiety and Insomnia		Social Dysfunction		Depression	
SCORE	GHQ28	CGHQ28	GHQ28	CGHQ28	GHQ28	CGHQ28	GHQ28	CGHQ28
**0**	56.9	3.9	60.8	7.8	74.5	2.0	92.2	64.7
**1**	11.8	7.8	13.7	9.8	11.8	2.0	3.9	15.7
**2**	7.8	19.6	2.0	15.7	3.9	2.0	2.0	5.9
**3**	7.8	5.9	5.9	9.8	0	3.9	0	3.9
**4**	3.9	17.6	9.8	15.7	2.0	2.0	2.0	2.0
**5**	2.0	9.8	5.9	13.7	5.9	2.0	0	5.9
**6**	3.9	15.7	2.0	9.8	0	13.7	0	2.0
**7**	5.9	19.6	0	17.6	2.0	72.5	0	0
**Total**	100	100	100	100	100	100	100	100

Notes: GHQ-28 coded 0-0-1-1 (case ≥ 6); CGHQ-28 coded 0-1-1-1 (case ≥ 13). Higher scores indicate worse health.

**Table 4:** Scores of chronic health problems (CGHQ-28) of subscales, and comparison among variables.

			Sex		Marital Status		Professional Role		Profession		
	Total		Male	Female	Married	Unmarried	Emergency Team or Coordination	Both	Physician	Nurse	EMT ^a^
	n	51	30	21	32	19	27	24	23	16	12
***Somatic*** ***Symptoms***	**Mean** **(SD)**	4.16 (2.18)	3.83 (2.09)	4.62 (2.27)	4.25 (2.18)	4 (2.21)	4 (2.39)	4.33 (1.95)	4.26 (1.96)	4.38 (2.8)	3.67 (1.67)
	***p***	-	-	0.187	-	0.657	-	0.674	-	-	0.547
***Anxiety and Insomnia***	**Mean** **(SD)**	3.84 (2.25)	3.57 (2.03)	4.24 (2.53)	4.09 (2.25)	3.42 (2.24)	3.48 (2.29)	4.25 (2.17)	4.17 (2.19)	3.75 (2.54)	3.33 (2.02)
	***p***	-	-	0.242	-	0.297	-	0.209	-	-	0.532
***Social*** ***Dysfunction***	**Mean** **(SD)**	6.25 (1.64)	6.33 (1.35)	6.14 (2.01)	6.13 (1.66)	6.47 (1.61)	6 (1.94)	6.54 (1.18)	6.52 (1.2)	5.44 (2.37)	6.83 (0.39)
	***p***	-	-	0.751	-	0.068	-	0.279	-	-	0.12
***Depression***	**Mean** **(SD)**	0.88 (1.58)	0.73 (1.41)	1.1 (1.81)	1 (1.72)	0.68 (1.34)	0.63 (1.18)	1.17 (1.93)	1 (1.78)	1 (1.79)	0.5 (0.67)
	***p***	-	-	0.605	-	0.599	-	0.535	-	-	0.992
***Global score***	**Mean** **(SD)**	15.14 (5.36)									

^a^ EMT: emergency medical technician.

**Table 5:** Mean (SD) COPSOQ-ISTAS21 scores for each psychosocial dimension by sex, marital status, shift type, and professional group.

		Sex			Marital Status			Professional Role			Profession			
	Total	Male	Female		Married	Unmarried		Emergency Team or Coordination	Both		Physician	Nurse	EMT ^a^	
	Mean (SD)	Mean (SD)		*p*	Mean (SD)		*p*	Mean (SD)		*p*	Mean (SD)			*p*
***Psychological*** ***Demands***	14.12 (2.74)	14.13 (2.85)	14.1 (2.64)	0.962	14 (2.53)	14.32 (3.13)	0.695	13.74 (2.89)	14.54 (2.55)	0.302	14.39 (2.5)	13.88 (3.34)	13.92 (2.47)	0.817
***Active Job*** ***Development***	21.06 (4.7)	20.57 (4.82)	21.76 (4.54)	0.376	21.38 (4.78)	20.53 (4.62)	0.538	20.63 (3.63)	21.54 (5.71)	0.494	21.78 (7.71)	20.25 (3.21)	20.75 (4.34)	0.594
***Insecurity***	5.61 (3.9)	6.17 (3.89)	4.81 (3.87)	0.225	5.69 (3.44)	5.47 (4.67)	0.852	5.33 (3.99)	5.92 (3.86)	0.599	6.17 (3.73)	4.44 (4.41)	6.08 (3.45)	0.356
***Social Support*** ***Leadership***	24.53 (6.3)	23.77 (6.61)	25.62 (5.82)	0.306	26.25 (4.64)	21.63 (7.7)	0.025 *	24.22 (7.05)	24.88 (5.47)	0.716	25.26 (5.25)	22.19 (7.96)	26.25 (5.19)	0.183
***Double Presence***	8.24 (4.03)	7.67 (3.6)	9.05 (4.54)	0.232	8.31 (3.7)	8.11 (4.56)	0.861	7.3 (4.28)	(9.29) (3.53)	0.078	9.17 (3.56)	8.63 (4.33)	5.92 (3.87)	0.066
***Esteem***	7.53 (3.49)	7.57 (3.53)	7.48 (3.52)	0.928	8.59 (2.78)	5.74 (3.89)	0.04 *	(7.19) (3.97)	(7.92) (2.89)	0.460	7.96 (2.95)	6.69 (3.89)	7.83 (3.97)	0.514

* *p* ≤ 0.05. ^a^ EMT: emergency medical technician. Values are raw COPSOQ-ISTAS21 scores coded according to the Spanish ISTAS21 manual. In some dimensions, higher scores reflect greater exposure (e.g., Psychological Demands, Insecurity, Double Presence), whereas in others they indicate more favorable job resources (Active Job/Development, Social Support/Leadership, Esteem). Risk interpretation in the text is based on the corresponding favorable, intermediate, and unfavorable exposure categories derived from these cut-offs.

## Data Availability

The data underlying this study contain potentially identifiable information about a small group of healthcare professionals and are therefore subject to restrictions imposed by the EU General Data Protection Regulation (GDPR) and Spanish data protection law, as well as by the conditions of the approval granted by the Regional Research Ethics Committee. For these reasons, the dataset is not publicly available. A suitably anonymized minimal dataset may be made available from the corresponding author upon reasonable request and subject to any additional institutional and regulatory requirements.

## Supplementary Materials

The following supporting information can be downloaded at: https://www.mdpi.com/article/10.3390/healthcare14010041/s1.

## Abbreviations

The following abbreviations are used in this manuscript:

061HEC	061 Health Emergency Center
CGHQ-28	Chronic General Health Questionnaire (28-item)
COPSOQ-ISTAS21	Copenhagen Psychosocial Questionnaire—Spanish ISTAS21 adaptation
EMT	Emergency Medical Technician (Spanish “técnico en emergencias sanitarias”)
