# Supplementary material for: General Health (GHQ-28/CGHQ-28) and Psychosocial Risks (COPSOQ-ISTAS21) in Prehospital Emergency Professionals: A Pre-COVID-19 Cross-Sectional Study in Southern Spain
Source: Healthcare (Basel). 2025 Dec 23;14(1):41. doi: 10.3390/healthcare14010041 (PMC12786040; doi:10.3390/healthcare14010041)
Supplement: Supplementary file 1 [file healthcare-14-00041-s001.zip › healthcare-4037149-SI.pdf]

healthcare

MDPI

Supplementary Table S1. Spearman's rank correlations between the GHQ-28 and the CGHQ-28 subscales. Coefficients (ϱ), two-tailed significance values, and sample size (n = 51) are reported for all comparisons. *Statistically significant correlations ($p < 0.05$).

GHQ-28 / CGHQ-28	Somatic Symptoms CGHQ	Anxiety and Insomnia CGHQ	Social Dysfunction CGHQ	Depression CGHQ	Total CGHQ
**Somatic Symptoms GHQ**	0.526 (p=0.000*)	0.417 (p=0.002*)	–0.044 (p=0.760)	0.324 (p=0.020*)	0.480 (p=0.000*)
**Anxiety and Insomnia GHQ**	0.360 (p=0.010*)	0.568 (p=0.000*)	–0.027 (p=0.853)	0.463 (p=0.001*)	0.541 (p=0.000*)
**Social Dysfunction GHQ**	0.222 (p=0.117)	0.338 (p=0.015*)	0.099 (p=0.487)	0.431 (p=0.002*)	0.384 (p=0.005*)
**Depression GHQ**	0.149 (p=0.297)	0.284 (p=0.043*)	0.043 (p=0.763)	0.491 (p=0.000*)	0.396 (p=0.004*)
**Total GHQ**	0.462 (p=0.001*)	0.505 (p=0.000*)	0.035 (p=0.807)	0.435 (p=0.001*)	0.552 (p=0.000*)

Supplementary Table S2. Spearman's rank correlations between the GHQ-28 dimensions and the psychosocial factors of the COPSOQ-ISTAS21. The table includes correlation coefficients (ϱ), two-tailed p-values, and sample size (n = 51). No statistically significant associations were observed between the GHQ-28 and COPSOQ-ISTAS21 dimensions.

GHQ-28 / COPSOQ-ISTAS21	Psychological Demands	Active Job Development	Insecurity	Social Support Leadership	Double Presence	Esteem
**Somatic Symptoms**	0.152 (p=0.286)	0.043 (p=0.767)	0.038 (p=0.794)	0.056 (p=0.697)	0.204 (p=0.151)	–0.124 (p=0.386)
**Anxiety and Insomnia**	0.233 (p=0.100)	–0.082 (p=0.568)	0.156 (p=0.275)	–0.175 (p=0.219)	0.249 (p=0.078)	–0.140 (p=0.326)
**Social Dysfunction**	0.128 (p=0.370)	0.024 (p=0.865)	0.124 (p=0.385)	–0.128 (p=0.370)	0.127 (p=0.376)	–0.078 (p=0.588)
**Depression**	0.048 (p=0.740)	0.126 (p=0.378)	–0.174 (p=0.223)	–0.064 (p=0.657)	0.247 (p=0.081)	0.048 (p=0.736)
**Total**	0.200 (p=0.160)	0.030 (p=00.836)	0.101 (p=0.481)	–0.060 (p=0.675)	0.228 (p=0.108)	–0.118 (p=0.411)

Supplementary Table S3. Spearman's rank correlations between the CGHQ-28 dimensions and the psychosocial factors of the COPSOQ-ISTAS21. Correlation coefficients (ϱ), two-tailed significance levels, and sample size (n = 51) are presented0. *Statistically significant correlations ($p < 0.05$).

CGHQ-28 /COPSOQ-ISTAS21	Psychological Demands	Active Job Development	Insecurity	Social Support Leadership	Double Presence	Esteem
**Somatic Symptoms**	0.071 (p=0.619)	−0.004 (p=0.978)	0.065 (p=0.651)	−0.025 (p=0.864)	0.273 (p=0.053)	−0.063 (p=0.659)
**Anxiety and Insomnia**	0.203 (p=0.153)	−0.072 (p=0.615)	0.150 (p=0.292)	−0.208 (p=0.144)	0.336 (p=0.016*)	−0.085 (p=0.552)
**Social Dysfunction**	−0.077 (p=0.593)	−0.021 (p=0.881)	0.000 (p=10.000)	−0.125 (p=0.381)	0.042 (p=0.767)	−0.101 (p=0.482)
**Depression**	0.016 (p=0.913)	0.229 (p=0.106)	−0.088 (p=0.538)	0.015 (p=0.918)	0.154 (p=0.279)	−0.093 (p=0.516)
**Total**	0.144 (p=0.312)	−0.037 (p=0.795)	0.033 (p=0.817)	−0.163 (p=0.253)	0.312 (p=0.026*)	−0.114 (p=0.424)

https://doi.org/10.3390/healthcare14010041
